# The *Drosophila* Translational Control Element (TCE) Is Required for High-Level Transcription of Many Genes That Are Specifically Expressed in Testes

**DOI:** 10.1371/journal.pone.0045009

**Published:** 2012-09-11

**Authors:** Rebeccah J. Katzenberger, Elizabeth A. Rach, Ashley K. Anderson, Uwe Ohler, David A. Wassarman

**Affiliations:** 1 University of Wisconsin School of Medicine and Public Health, Department of Cell and Regenerative Biology, Madison, Wisconsin, United States of America; 2 Institute for Genome Sciences and Policy, Departments of Biostatistics and Bioinformatics and Computer Science, Duke University, Durham, North Carolina, United States of America; University of Birmingham, United Kingdom

## Abstract

To investigate the importance of core promoter elements for tissue-specific transcription of RNA polymerase II genes, we examined testis-specific transcription in *Drosophila melanogaster*. Bioinformatic analyses of core promoter sequences from 190 genes that are specifically expressed in testes identified a 10 bp A/T-rich motif that is identical to the translational control element (TCE). The TCE functions in the 5′ untranslated region of *Mst(3)CGP* mRNAs to repress translation, and it also functions in a heterologous gene to regulate transcription. We found that among genes with focused initiation patterns, the TCE is significantly enriched in core promoters of genes that are specifically expressed in testes but not in core promoters of genes that are specifically expressed in other tissues. The TCE is variably located in core promoters and is conserved in *melanogaster* subgroup species, but conservation dramatically drops in more distant species. In transgenic flies, short (300–400 bp) genomic regions containing a TCE directed testis-specific transcription of a reporter gene. Mutation of the TCE significantly reduced but did not abolish reporter gene transcription indicating that the TCE is important but not essential for transcription activation. Finally, mutation of testis-specific TFIID (tTFIID) subunits significantly reduced the transcription of a subset of endogenous TCE-containing but not TCE-lacking genes, suggesting that tTFIID activity is limited to TCE-containing genes but that tTFIID is not an obligatory regulator of TCE-containing genes. Thus, the TCE is a core promoter element in a subset of genes that are specifically expressed in testes. Furthermore, the TCE regulates transcription in the context of short genomic regions, from variable locations in the core promoter, and both dependently and independently of tTFIID. These findings set the stage for determining the mechanism by which the TCE regulates testis-specific transcription and understanding the dual role of the TCE in translational and transcriptional regulation.

## Introduction

The core promoter is the region surrounding the transcription start site (TSS) of a gene that functions to recruit RNA polymerase II to initiate transcription [1]. Historically, core promoters were thought to function by a single generic mechanism. However, recent discoveries suggest that core promoters function by numerous different mechanisms and that the differences are important for tissue-specific transcription programs [2], [3]. The first key discovery is the diversity of elements that contribute to core promoter function [4]–[7]. There are no universal core promoter elements. Familiar core promoter elements, such as the TATA box and initiator (Inr), are only present in a minority of promoters, and computational analyses, followed by experimental validation, have helped to identify an ever-increasing number of core promoter elements, such as the motif ten element (MTE) [4], [8]. The observation that maternally expressed genes in *Drosophila* have different core promoter element compositions than zygotically active genes highlights the importance of core promoter element diversity for transcription regulation [5], [9].

The second key discovery is the existence of two classes of transcription initiation patterns that are associated with different types of core promoter elements and chromatin structures [1], [9]. Genes with focused initiation patterns initiate transcription at a single nucleotide or within a region of several nucleotides, and genes with dispersed initiation patterns initiate transcription at numerous sites in a region of 100–200 bp. Focused initiation patterns tend to be associated with location-specific core promoter elements, such as a TATA box at −30 bp or an Inr at −2 bp relative to the TSS, and are generally associated with regulated genes. Dispersed initiation patterns tend to contain variably located core promoter elements, such as the DNA replication-related element (DRE), and are generally associated with constitutively expressed genes [9], [10].

The third key discovery is the diversity of protein factors that recognize core promoter elements. The general transcription factor TFIID is the major core promoter recognition factor [11]. TFIID is a multi-protein complex composed of TATA-binding protein (TBP) and ∼15 TBP-associated factors (TAFs) [12], [13]. Recognition of core promoters by TFIID is exemplified by TBP, which binds the TATA box, and TAF1 and TAF2, which together bind the Inr [1], [3]. In metazoans, paralogs of TBP, such as TBP-related factor 2 (TRF2), and TAFs, such as TAF4b, play unique roles in regulating transcription [14], [15]. Thus, combinatorial capacity, provided by diverse core promoter elements and TFIID subunits, is likely to play a major role in tissue-specific transcription.

*Drosophila* spermatogenesis is an ideal system to investigate roles for core promoter element and TFIID diversity in the regulation of tissue-specific transcription. Genome-wide analyses of mRNA expression in a variety of *Drosophila* tissues have determined that >1,000 genes are preferentially or uniquely expressed in testes [16], [17]. Transcription of genes that are specifically expressed in testes is commonly controlled by 100–400 bp genomic regions that include the TSS [18], [19]. Particular sequences have been identified that are important for the testis-specific transcription of genes; however, no common testis-specific core promoter elements have been identified. In regard to TFIID diversity, paralogs of ubiquitously expressed TAF4, TAF5, TAF6, TAF8, and TAF12 (No hitter (Nht), Cannonball (Can), Meiosis I arrest (Mia), Spermatocyte arrest (Sa), and Ryan express (Rye), respectively) are predominantly expressed in testes [19]–[21]. Flies mutant for any one of the testis-specific TAFs (tTAFs) are male sterile, they arrest spermatogenesis prior to meiosis, and they have reduced transcription of genes required for entry into meiosis. tTAFs may be components of a testis-specific TFIID (tTFIID) complex since they co-localize within spermatocyte nuclei [22], [23].

Based on these findings, we hypothesized that *Drosophila* genes that are specifically expressed in testes contain novel core promoter elements that regulate testis-specific transcription. Furthermore, we hypothesized that testis-specific core promoter elements are recognized by a tTFIID complex. Here, we present experiments that test these hypotheses.

## Results

### Identification of the TCE as a testis-specific core promoter element

To identify sequence elements that are enriched in core promoters of genes that are specifically expressed in testes, we examined core promoters surrounding TSSs with testis-specific activity. While recent sequencing protocols have made it possible to map TSSs at high resolution and throughput, such approaches are not straightforward to apply to small amounts of RNA from specific tissues. We therefore used a high quality set of 5,665 TSSs for 3,990 genes, which had been defined by hierarchical clustering of expressed sequence tags (ESTs) from 15 different *Drosophila melanogaster* libraries [9]. Shannon entropy was used as a metric to identify 1,997 condition-specific TSSs; those that occurred specifically in one of eight cDNA libraries with sufficient coverage (embryo, larva/pupa, head, ovary, testis, Schneider cell (S2), mbn2 hemocytic cell, and fat body). Among the 1,997 TSSs, 1,395 had focused initiation patterns. Of these, 190 were specifically expressed in testes and 1,205 were specifically expressed in another condition.

**Figure 1:**
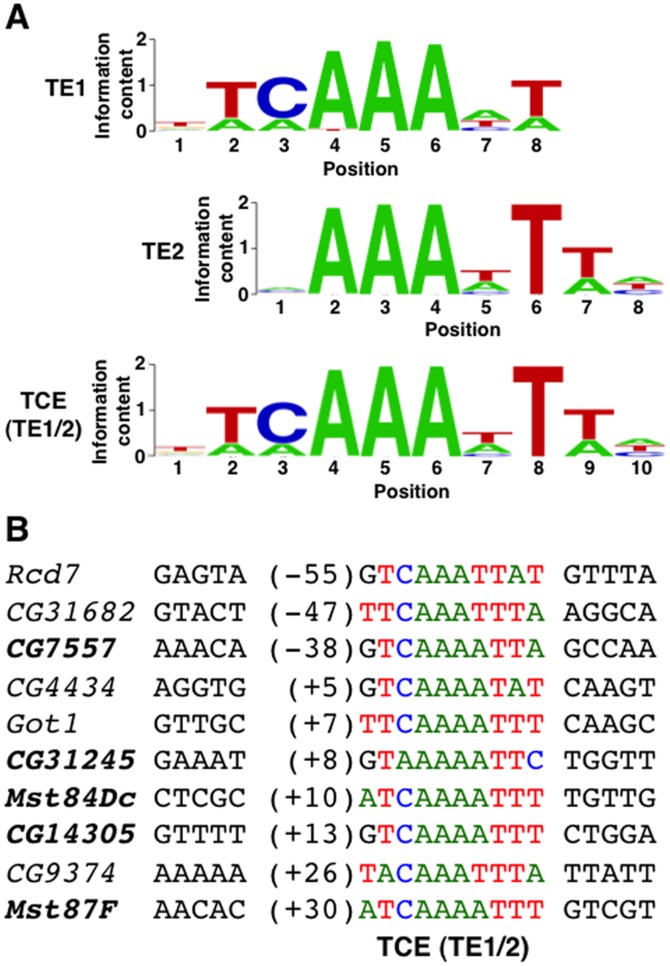
The TCE (TE1/2) and related sequences, TE1 and TE2. (A) Shown are DNA sequence logos of TE1, TE2, and TE1/2, which is identical to the previously defined TCE [29]. The logos have been aligned relative to the triplet adenines. (B) *Drosophila* genes that are specifically expressed in testes are aligned by the TCE. Genes indicated in bold font were subject to further analysis in Figure 4 and Table S3. Indicated in parentheses is the TCE location relative to the TSS. TCE nucleotides are colored to match panel A.

Computational sequence motif searches by MEME and Gibbs Sampling identified sequence elements that were overrepresented in the vicinity of the 190 testis-specific TSSs [24], [25]. For these searches, core promoters were defined as the region from 60 bp upstream (−60) to 40 bp downstream (+40) of the TSS. Details of the search parameters are provided in the Materials and Methods. The independent searches returned highly similar results; two adenine/thymine (A/T)-rich elements were found to occur in ∼60% of the testis-specific core promoters. DNA sequence logo representations of the elements identified by Gibbs Sampling are shown in Figure 1A and position frequency matrices for the elements are shown in Tables S1 and S2 [26]. The elements were named Testis Element 1 (TE1) and Testis Element 2 (TE2).

To determine the extent to which the elements are testis-specific, Patser was used to scan for matches of TE1 and TE2 in core promoters from each of the eight conditions [27]. This analysis revealed that TE1 and TE2 were present in 46% and 49% of the testis-specific core promoters, respectively (Table 1). At the same false positive stringency (i.e. at matrix scores corresponding to the same P-values), the frequency of TE1 or TE2 in testis-specific core promoters was higher than either the TATA box (33%) or Inr (40%) in embryo-specific core promoters [28]. In the sets of other condition-specific promoters, TE1 and TE2 occurred at a noticeably lower rate (9–11% in head and embryo promoters, for which the datasets were sufficiently large for analysis). These data indicate that TE1 and TE2 are overrepresented in testis-specific core promoters.

**Table 1:** The TCE (TE1/2) is enriched in testis-specific core promoters.

Condition	Specific TSSs	TE1	TE2	TCE (TE1/2)
Testis	190	87 (46%)	93 (49%)	85 (45%)
Embryo	765	71 (9%)	85 (11%)	79 (10%)
Head	370	38 (10%)	33 (9%)	34 (9%)
fat body	6	1 (17%)	1 (17%)	1 (17%)
larva/pupa	22	2 (9%)	0 (0%)	1 (5%)
Ovary	7	0 (0%)	1 (14%)	0 (0%)
S2 cell	25	3 (12%)	6 (24%)	4 (16%)
mbn2 cell	10	2 (20%)	0 (0%)	1 (10%)
Total	1395	204 (15%)	219 (16%)	205 (15%)

As illustrated in Figure 1A, both TE1 and TE2 had three consecutive invariant adenines, raising the possibility that TE1 and TE2 overlap to form a longer element, which was named Testis Element 1/2 (TE1/2). Indeed, 45% of testis-specific core promoters were found to contain TE1/2, a 10 nt position weight matrix (PWM) joined from TE1 and TE2 (Figure 1A and Table 1). Figure 1B provides examples of TE1/2 sequences in the core promoters of genes that are specifically expressed in testes. Thus, we consider TE1 and TE2 motifs non-canonical TE1/2 motifs rather than as separate or distinct functional motifs.

TE1/2 is identical to a previously defined motif called the Translational Control Element (TCE) [29]. For this reason, we hereafter refer to TE1/2 as the TCE. The TCE is a 12 nt motif (ACATCNAAATTT) that was defined based on sequence comparison among seven members of the *Mst(3)CGP* gene family, including *Mst(3)87F*, that are specifically expressed in testes. In *Mst(3)CGP* genes, the TCE is invariantly located at+28 relative to the TSS. A reporter gene containing a fragment of *Mst(3)87F* from −670 to+51 fused upstream of the *LacZ* gene was used to demonstrate that the TCE functions as a repressor of translation in diploid stage cells of third instar larval testes. Deletion or mutation of the TCE in the reporter gene results in premature translation, as observed by ß-galactosidase (ß-gal) activity in third instar larval testes. Additionally, moving the TCE 26 nt downstream to+54 results in premature translation, indicating that the TCE regulates translation in a location-specific manner [30]. However, it has been noted that other genes that are specifically expressed in testes and whose expression is under translational control contain TCE-like sequences at positions other than+28, bringing into question the location-specific requirement for activity [31]. Finally, there is limited evidence that the TCE regulates transcription in testes. Insertion of the TCE at+28 restores transcriptional activity to *ß2-tubulin* (*ß2t*) transgenes that lack a 14 bp fragment required for testis-specific transcription. In subsequent studies, we investigated the role of the TCE in testis-specific transcriptional regulation.

**Figure 2:**
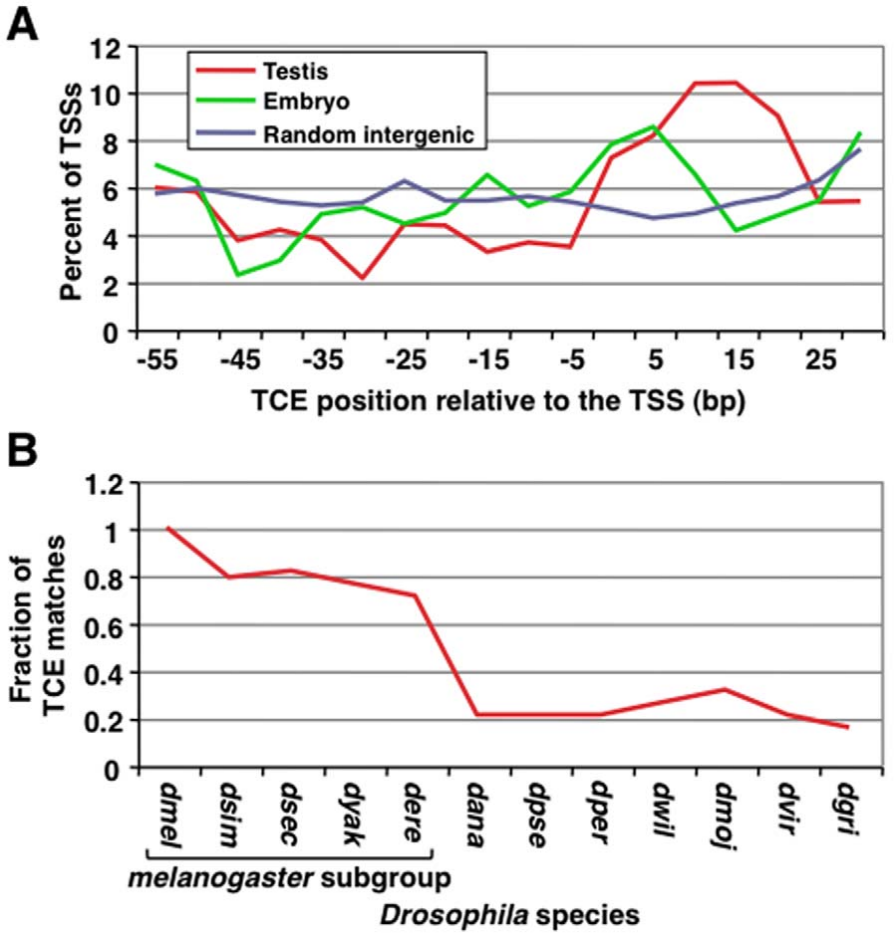
TCEs are variably located in testis-specific core promoters and are conserved in *melanogaster* subgroup species. (A) A smoothed plot of the location of the TCE in testis-specific core promoters, embryo-specific core promoters, and random intergenic regions, which serve as background. The calculated background is similar to the expected background of 5.6 (100/18 bins of 5-nt each). Plotted along the x-axis is the location of the TCE relative to the TSS. Plotted along the y-axis is the fraction of genes with a TCE located within a 5-nt interval. (B) Plotted is the TCE conservation across 12 *Drosophila* species, as determined by the fraction of matches in the −5/+25 region in *D. melanogaster* as seen in panel A that are also found at corresponding locations in cross-species alignments. *Drosophila* species abbreviations are as follow: *dmel* (*melanogaster*), *dsim* (*simulans*), *dsec* (*sechellia*), *dyak* (*yakuba*), *dere* (*erecta*), *dana* (*ananassae*), *dpse* (*pseudoobscura*), *dper* (*persimilis*), *dwil* (*willistoni*), *dmoj* (*mojavensis*), *dvir* (*virilis*), and *dgri* (*grimshawi*).

Since genes with focused initiation patterns typically have location-specific core promoter elements, we examined the location of the TCE in core promoters of genes that are specifically expressed in testes. TCE matches were located throughout the −60 to+40 core promoter region but were enriched in the −5 to+25 region (Figure 2A). In contrast, random intergenic sequences showed no such pattern, and enrichments were lower in the set of core promoters from genes specifically expressed in embryos. Thus, among genes specifically expressed in testes, the TCE is not strictly located within core promoters but is preferentially located at and immediately downstream of the TSS.

Overall, the evolutionary conservation level of TCEs located in the −5/+25 region is ∼75% in *melanogaster* subgroup species and drops to ∼25% in more distant species (Figure 2B). The sharp decline in conservation outside of the *melanogaster* subgroup was not unexpected, as the level of preferential conservation is comparable to other variably located core promoter elements, such as the DRE [9], [10]. These data suggest that testis-specific core promoters for species outside of the *melanogaster* subgroup either contain TCEs at different locations or contain different elements.

### The TCE is necessary for the transcription of a subset of genes that are specifically expressed in testes

**Figure 3:**
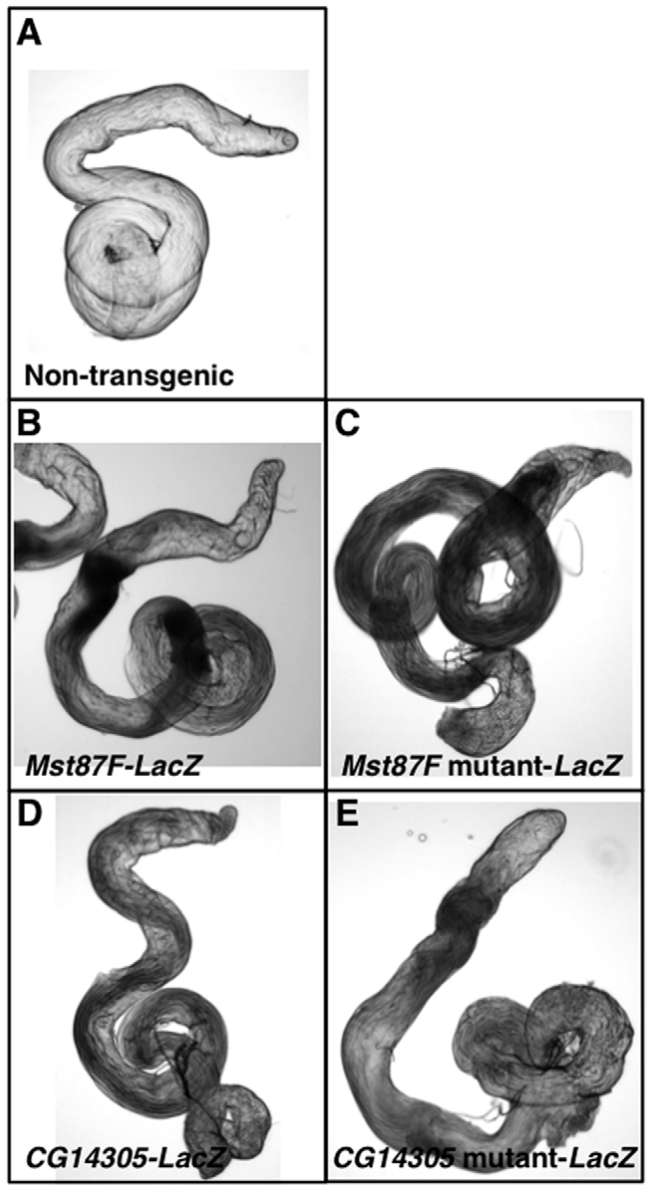
*Mst87F* and *CG14305* promoter regions direct expression in testes. Shown is ß-gal activity staining of testes from (A) a non-transgenic fly, (B) an *Mst87F-LacZ* transgenic fly, (C) an *Mst87F* mutant-*LacZ* transgenic fly, (D) a *CG14305*-*LacZ* transgenic fly, and (E) a *CG14305* mutant-*LacZ* transgenic fly. Staining was performed under identical reaction conditions.

To examine the importance of the TCE for testis-specific transcription, we focused our studies on five TCE-containing genes that are specifically expressed in testes, *Mst84Dc*, *Mst87F*, *CG7557*, *CG14305*, and *CG31245*, two of which (*Mst84Dc* and *Mst87F*) are members of the *Mst(3)CGP* gene family. These genes were chosen because they contain TCEs at different locations relative to the TSS and they differed in mRNA expression level in testes (Figure 1B and Table S3). To determine the extent to which genomic regions encompassing TCE-containing core promoters are sufficient to direct testis-specific transcription, transgenic reporter flies were generated. Short (300–400 bp) genomic regions (−182 to+145 for *Mst84Dc*, −104 to+187 for *Mst87F*, −218 to+138 for *CG7557*, −199 to+209 for *CG14305*, and −224 to+177 for *CG31245*) were cloned directly upstream of the *LacZ* coding region in a transformation vector that can be targeted to specific genomic sites by the phiC31 integrase system [32]. To eliminate position effects, all of the transgenes were targeted to the identical site at 86F on the third chromosome. *LacZ* expression was examined in testes of transgenic flies by a whole mount assay for ß-gal activity. ß-gal activity was detected in testes of transgenic reporter lines but not non-transgenic lines (Figure 3A, B, and D). Thus, short genomic regions containing a TCE are sufficient to direct transcription in testes.

Quantitative real-time reverse transcription-PCR (qPCR) was used to determine whether *LacZ* expression was testis-specific. *ß-gal* mRNA level relative to *actin* mRNA level was determined for RNA extracted from adult flies: whole males, whole females, testes, and male carcasses (the tissue remaining after testis dissection). This analysis revealed that reporters for all five genes were highly expressed in males relative to females (Table 2). Moreover, reporters for all of the genes were significantly enriched in testes relative to male carcasses. These results are consistent with those from a prior study of *Mst87F* transcription regulatory sequences [33]. Thus, short genomic regions encompassing the TCE and the TSS are sufficient to direct testis-specific transcription.

**Table 2:** TCE-containing genomic regions direct testis-specific transcription.

Gene	Testis	Male carcass	Whole male	Whole female	male/female
*Mst84Dc*	2.435±0.268	0.005±0.001	0.699±0.037	0.005±0.001	140
*Mst87F*	1.681±0.070	0.005±0.001	0.114±0.040	0.003±0.001	38
*CG7557*	0.506±0.030	0.003±0.000	0.130±0.016	0.002±0.000	65
*CG14305*	0.348±0.007	0.005±0.000	0.032±0.005	0.002±0.001	16
*CG31245*	0.457±0.049	0.007±0.001	0.145±0.015	0.005±0.000	29

**Figure 4:**
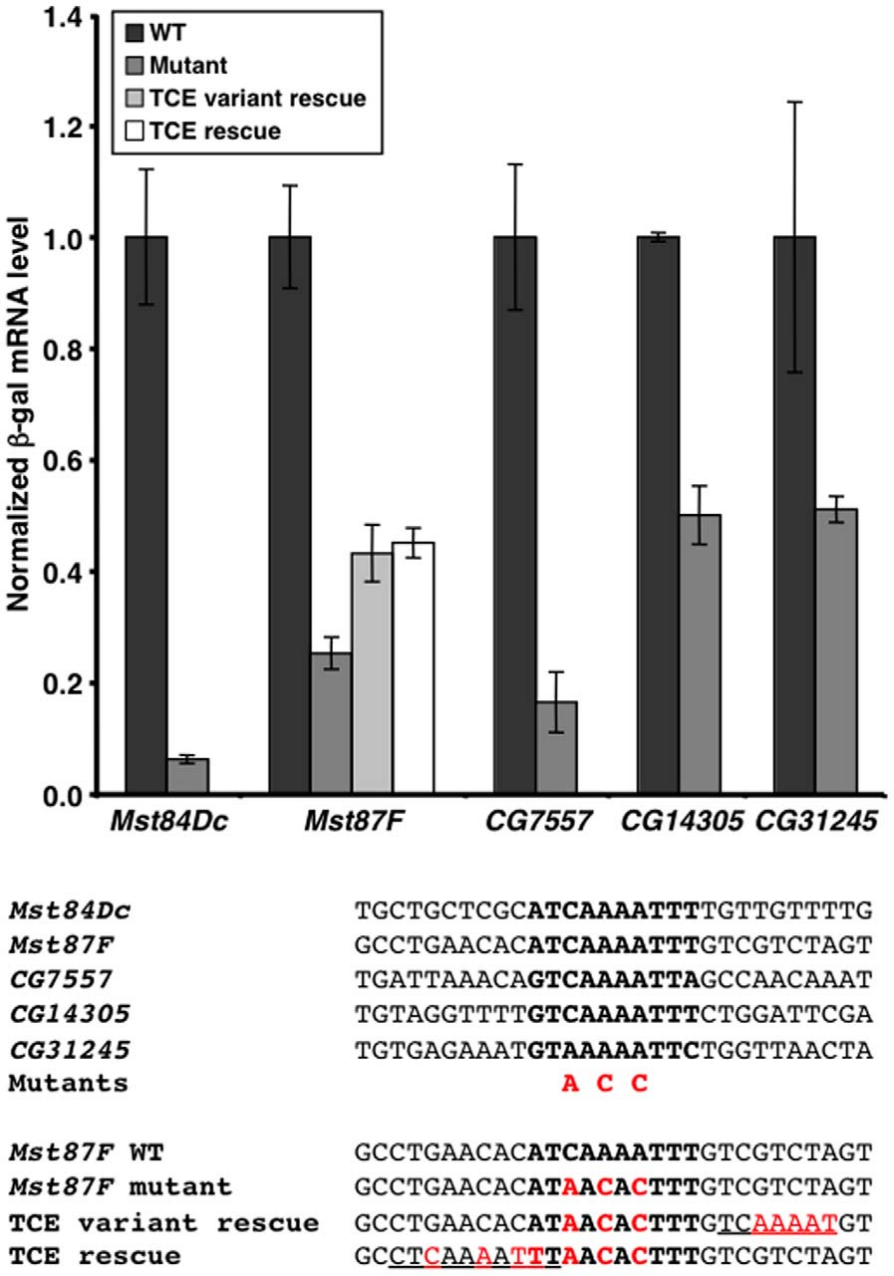
TCE mutations reduce the transcription of genes that are specifically expressed in testes. Graphed is the mRNA expression of *ß-gal* relative to *actin* in the testes of transgenic flies. Wildtype (WT) and TCE mutant transgenes were examined for each of the indicated genes. Additionally, for *Mst87F*, two rescue transgenes, containing ectopic TCE motifs in the context of a mutant TCE sequence, were examined. mRNA levels were normalized to those of the wildtype transgene. Statistically significant differences are indicated in the text. Below the graph are wildtype and mutant TCE sequences for the five genes analyzed in transgenic reporter gene assays, as well as the *Mst87F* wild type, mutant, and rescue sequences that were used in the transgenic reporter gene assays. Indicated in red are mutated nucleotides. Underlined are the ectopic TCE motifs.

The qPCR transgenic reporter assay was used to determine the extent to which the TCE is necessary for testis-specific transcription of the genes analyzed in Table 2. Site-directed mutagenesis was used to introduce three point mutations into the TCE for each of the five *LacZ* reporter genes. For example, the *Mst87F* TCE sequence ATCAAAATTT was mutated to ATAACACTTT (Figure 4, bottom). Transgenic flies were generated with the reporter genes inserted at 86F on the third chromosome, so that they could be directly compared to the wildtype transgenes. ß-gal activity in testes was not overtly different between wildtype and mutant transgenic flies; however, the assay is not sensitive to small changes in expression (Figure 3). To quantitatively assay transcription, qPCR was used to determine *ß-gal* mRNA levels relative to *actin* mRNA levels for RNA extracted from testes of transgenic flies. This analysis revealed that mutation of the TCE significantly reduced expression of the *Mst84Dc*, *Mst87F*, *CG7557*, and *CG14305* transgenes (P<0.05) (Figure 4, top). The average steady-state mRNA level was reduced to 6.2%, 25.2%, 16.4%, and 50.0% of normal levels in *Mst84Dc*, *Mst87F*, *CG7557*, and *CG14305* mutants, respectively. Mutation of the *CG31245* transgene reduced expression to 51.2% of normal levels, but the effect did not meet the significance cutoff (P = 0.07). In accord with the documented role of the TCE in translational repression, the lack of a change in ß-gal protein expression in TCE mutant flies (Figure 3) may be due to compensatory decreases and increases in *ß-gal* gene transcription and translation, respectively [29]–[31]. Reduced mRNA expression caused by the TCE mutation could be due to reduced gene transcription or increased mRNA decay. However, an effect on mRNA decay is unlikely because, for genes such as *CG7557*, the TCE is located upstream of the TSS and is not transcribed and, thus, cannot directly serve as a regulatory element for mRNA decay. Therefore, the TCE is necessary for activating the transcription of a subset of genes that are specifically expressed in testes.

To further examine the role of TCE in testis-specific transcription, we determined the extent to which introduction of a TCE motif at a new location within the core promoter could rescue the transcription defect caused by mutation of the normal TCE motif. Site-directed mutagenesis was used to introduce the TCE sequence CTCAAAATTT immediately upstream of the mutant TCE sequence in the *LacZ* reporter gene for *Mst87F* (Figure 4, bottom). The reporter gene was then inserted at 86F on the third chromosome, so that it could be directly compared to the wildtype and the TCE mutant transgenes. qPCR was used to determine *ß-gal* mRNA level relative to *actin* mRNA level for RNA extracted from testes of transgenic flies. This analysis revealed that introduction of the TCE motif caused a significant ∼2-fold increase in transcription of the mutant reporter gene (P<0.05) (Figure 4, top). Similarly, introduction of a variant TCE sequence GTCAAAAT immediately downstream of the mutant TCE caused a significant ∼2-fold increase in the transcription of the mutant reporter gene (P<0.05) (Figure 4, top and bottom). These data provide additional support for a role for the TCE in the activation of testis-specific transcription.

### tTAFs are required for the transcription of a subset of TCE-containing genes that are specifically expressed in testes

**Figure 5:**
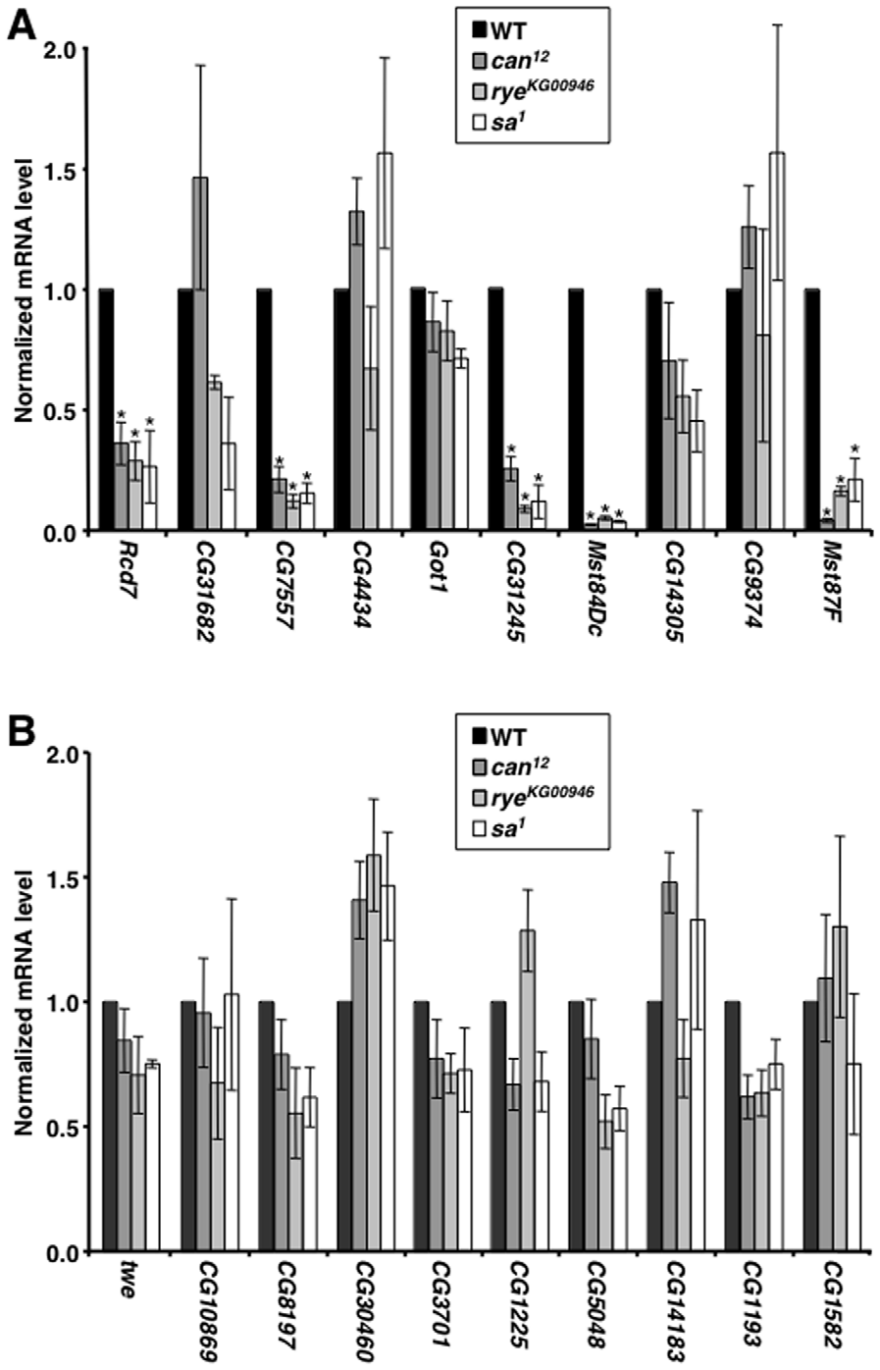
tTAFs regulate the transcription of a subset of TCE-containing genes. Graphed is the mRNA expression level for the indicated genes in testes of wildtype (WT) and mutant flies. Expression levels were normalized to *actin* and to the expression level in wildtype flies. Asterisks indicate significant (P<0.05) changes in expression level in mutant relative to wildtype flies as determined by one-way ANOVA in conjunction with Bonferroni post-tests. (A) Graphed is the relative transcription level of genes with focused initiation patterns that contain a TCE in the core promoter. (B) Graphed is the relative transcription level of genes with focused initiation patterns that do not contain a TCE in the core promoter. *twe* is specifically transcribed in testes and lacks a TCE but was not included in the 190 genes that are specifically expressed in testes.

Since TAF components of TFIID commonly bind core promoter elements, we hypothesized that tTAF components of tTFIID would be required for the transcription of TCE-containing genes that are specifically expressed in testes [3]. In fact, mutation of the tTAF *can* was previously shown to reduce *Mst87F* and *Mst84Dc* transcription in testes [20]. qPCR of RNA extracted from testes was used to examine the requirement of the tTAF genes *can*, *rye*, and *sa* for transcription of the ten TCE-containing genes listed in Figure 1B. Mutation of any of the tTAF genes significantly reduced the mRNA level of five of the genes, *Rcd7*, *CG7557*, *CG31245*, *Mst84Dc*, and *Mst87F* (P<0.05) (Figure 5A). In contrast, tTAF mutations did not affect the transcription of ten genes that lack a TCE, all of which, except *twine* (*twe*), were chosen from among the 190 genes that were used to identify the TCE (Figure 5B). Taken together, these data suggest an intricate functional relationship between the TCE and a tTFIID complex containing Can, Rye, and Sa.

## Discussion

Here, we have determined that the TCE is present in the core promoter of a substantial fraction of *Drosophila melanogaster* genes with focused transcription initiation patterns that are specifically expressed in testes. *In vivo* studies indicate that the TCE functions in a location-independent manner to activate the transcription of many genes that are specifically expressed in testes. Furthermore, *in vivo* studies indicate that a tTFIID complex containing tTAFs regulates testis-specific transcription of a subset of TCE-containing genes. To our knowledge, this is the first example in a metazoan organism of tissue-specific transcription regulation by a common, tissue-specific core promoter element.

Relatively little is known about *cis*-regulation of testis-specific transcription in *Drosophila*. A previous computational study applied a regression tree approach to explain sex-specific expression differences by the occurrence of co-occurring k-mers in 1 kb upstream regions but only identified female-specific significant sequence motifs [34]. Studies of several genes indicate that short genomic regions encompassing the TSS are sufficient to direct testis-specific transcription and that the combined activities of elements within these regions provide testis specificity and control transcription level [18], [19]. For example, a genomic region from −53 to+172 of the *ß2t* gene is sufficient to direct transcription in testes [35], [36]. A ß2UE1 element at −45 is necessary for testis-specific transcription, and two elements, ß2UE2 at −29 and ß2DE1 at+60, are necessary for high-level transcription. Our data are consistent with these findings. Short genomic regions containing the TCE were sufficient to direct testis-specific transcription, and the TCE was necessary for high-level transcription in testes. What is unique about the TCE, relative to previously described testis elements, including those in the *ß2t* gene, is that the TCE is present in many genes that are specifically expressed in testes. Among the 190 genes with focused initiation patterns, 85 contained a TCE. Common core promoter elements may also coordinate testis-specific transcription programs in mammals. As in *Drosophila*, many mouse and human genes are specifically expressed in testes and short genomic regions surrounding the TSS are sufficient to direct testis-specific transcription [37]–[39].

An unexpected but potentially important feature of the testis-specific transcription mechanism is the variable location of the TCE in core promoters. The variable location was unexpected because genes with focused initiation patterns, which were used to identify the TCE, tend to be associated with location-specific core promoter elements, such as the TATA box, Inr, DPE, and MTE [9]. Furthermore, TFIID complexes have only been shown to bind location-specific core promoter elements, such as the TATA box, Inr, DPE, and MTE [3]. However, the TCE is located throughout core promoters. Moreover, TCEs located either upstream or downstream of the TSS were necessary for testis-specific transcription, and insertion of a TCE at non-endogenous sites in a core promoter partially restored normal transcription levels to a TCE mutant core promoter. We cautiously note that for some genes that are specifically expressed in testes the location of the TSS and/or the designation of a focused initiation pattern may be inaccurate. In contrast to most mapped fly TSSs, testis-specific TSSs were based on 5′ EST libraries prepared without cap trapping. While the same stringency was required as in previous work, in which promoters from cap-trapped libraries were examined, we expect the majority of promoters to be close to the indicated TSS but not necessarily exactly match in all cases [9]. In addition, the prevalence of focused initiation patterns appeared higher than was later determined by high-throughput sequencing [28].

Different trans-acting factors presumably regulate the translational and transcriptional activities of the TCE. For genes specifically expressed in testes, the TCE functions as an RNA element in the 5′ untranslated region of mRNAs to repress translation and as a DNA element in the core promoter of genes to activate transcription. Testis extracts are competent to form RNA-protein and DNA-protein complexes with the TCE *in vitro*, but specific nucleic acid-binding factors have not been identified [30]. Data presented here has shed some light on the DNA-protein complex. The variable location of the TCE raises the possibility that the TCE is a short-range enhancer that is bound by a transcription activator protein rather than a core promoter element that is bound by a component of the basal transcription machinery. Indeed, the TCE is unlikely to serve as a binding site for tTFIID since all TCE-containing genes did not require tTAFs for high-level transcription. As a short-range enhancer, the TCE may function analogously to OVO binding sites that regulate the transcription of genes in the ovary [40]. The lack of a strict requirement for tTFIID predicts the existence of a location-specific core promoter element that is recognized by tTFIID and functions in partnership with TCE at a subset of TCE-containing genes to determine testis-specific transcription. The predicted element is likely to only function in partnership with the TCE since tTAF mutations did not affect the transcription of genes that lacked a TCE. Additionally, the predicted element is likely to be of low sequence complexity since it was not identified in the bioinformatic analyses that minimally required a motif size of 5 nt. Finally, it is likely that transcription of TCE-containing genes is dependent on epigenetic features of the genome, such as nucleosome occupancy and histone modification [41], [42]. Thus, the TCE is a component of a combinatorial control mechanism that determines testis-specific transcription.

## Materials and Methods

### Bioinformatic analyses

Methods for Expressed Sequence Tag (EST) filtering and clustering and TSS identification are described in Rach *et al.* (2009) [9]. Briefly, TSSs were called based on multiple 5′ ESTs that were consistent with the gene annotation, upstream of annotated start codon(s), and whose 5′ ends clusters in short genomic windows. Condition-specific assignments were made on a per promoter basis by evaluating the Shannon entropy for tag frequencies from the individual libraries.

Testis elements were identified by MEME v3.0.14 run on 100 nt long core promoter sequences, using the “zero or one occurrence” parameter and a motif size of 5–15 nt [24]. Additionally, BioProspector, a Gibbs sampling based motif finder, was used, as provided in the BEST suite [25]. The motif width was set to 8 nt, and sampling was repeated 10 times and allowed to return up to 25 motifs. Both motif finders were provided with non-testis promoters to estimate background nucleotide frequencies, and reported motifs were required to occur in at least 20 sequences. MEME searches resulted in two significant hits, TE1 (E<10^−42^) and TE2 (E<10^−7^). Gibbs sampling reported both elements in all 10 runs, with TE1 consistently found as the top element. TE1/2 was formed by concatenating positions 1–3 from TE1 and positions 2–8 from TE2.

To identify motif matches in different sets of promoters, Patser was run with a P-value threshold of 10^−3^, using background nucleotide frequencies obtained from promoters not specific for any particular condition [27]. Position frequency matrices are presented in Tables S1 and S2. *Mst87F* was identified in an earlier iteration of the analysis that used a larger set of promoters and is not included in the set of 190 genes that are specifically expressed in testes.

The extent of motif conservation was evaluated using the protocol in Rach *et al.* (2009) [9]. Orthologous promoters were taken from the study describing the sequencing and analysis of 12 related *Drosophila* species [43]. Only promoters with identifiable alignments across all 12 species were analyzed further, comprising a set of 137 testis promoters. Motif conservation was evaluated in a pairwise manner by assessing the presence of co-occurring matches in *D. melanogaster* and one of the other 11 species. Matches to motifs were first determined independently in the promoters of each species and then counted as conserved if they occurred within+/−5 nt of the aligned position corresponding to the match in *D. melanogaster*.

### Transgenic flies

To generate TCE-containing transgenes, short genomic regions containing a TCE, as specified in the text, were amplified from *Drosophila melanogaster* genomic DNA by PCR and cloned into the *pattB-LacZ* plasmid [32]. TCE mutant and rescue transgenes were generated by site-directed mutagenesis using the QuickChange kit (Stratagene). Transgenic flies were generated by Rainbow Transgenics. Flies were maintained on standard cornmeal/molasses medium at 25°C. Molecular analyses were carried out on flies that were homozygous for the transgenes. ß-gal staining of transgenic flies was performed as described by Glaser *et al.* (1986) [44].

### qPCR

For mRNA quantitation, total RNA was collected from 0–4 d whole flies or fly tissues or S2 cells, reversed transcribed, and analyzed by real-time PCR as described by Katzenberger *et al.* (2006) [45]. Sequences of qPCR primers are provided in Table S4. Experiments were performed for four independent samples. Real-time data was analyzed using the 2^−ΔCt^ method [46]. Unpaired *t* tests were performed using Prism 4.0c (Graphpad Software).

## Supporting Information

Table S1 **Position frequency matrix for TE1.**
(DOC)

Table S2 **Position frequency matrix for TE2.**
(DOC)

Table S3 **qPCR of testis-specific genes relative to** ***actin.***
(DOC)

Table S4 **Primer sequences (5′-to-3′).**
(DOC)
