# Supplementary material for: The Drosophila Translational Control Element (TCE) Is Required for High-Level Transcription of Many Genes That Are Specifically Expressed in Testes
Source: PLoS One. 2012 Sep 11;7(9):e45009. doi: 10.1371/journal.pone.0045009 (PMC3439415; doi:10.1371/journal.pone.0045009)
Supplement: Table S1 — Position frequency matrix for TE1. (DOC) [file pone.0045009.s001.doc]

**Table S1. Position frequency matrix for TE1**

|  | **1** | **2** | **3** | **4** | **5** | **6** | **7** | **8** |
| --- | --- | --- | --- | --- | --- | --- | --- | --- |
| **A** | 15.52 | 25.86 | 23.28 | 96.54 | 99.98 | 99.12 | 48.27 | 28.45 |
| **C** | 12.93 | 0.87 | 76.71 | 0.01 | 0.01 | 0.01 | 20.69 | 0.01 |
| **G** | 20.69 | 0.01 | 0.01 | 0.01 | 0.01 | 0.01 | 0.01 | 0.01 |
| **T** | 50.86 | 73.27 | 0.01 | 3.45 | 0.01 | 0.87 | 31.03 | 71.54 |
