# Supplementary material for: The Drosophila Translational Control Element (TCE) Is Required for High-Level Transcription of Many Genes That Are Specifically Expressed in Testes
Source: PLoS One. 2012 Sep 11;7(9):e45009. doi: 10.1371/journal.pone.0045009 (PMC3439415; doi:10.1371/journal.pone.0045009)
Supplement: Table S2 — Position frequency matrix for TE2. (DOC) [file pone.0045009.s002.doc]

**Table S2. Position frequency matrix for TE2**

|  | **1** | **2** | **3** | **4** | **5** | **6** | **7** | **8** |
| --- | --- | --- | --- | --- | --- | --- | --- | --- |
| **A** | 37.27 | 99.07 | 99.98 | 99.98 | 40.00 | 0.01 | 35.45 | 36.36 |
| **C** | 36.36 | 0.91 | 0.01 | 0.01 | 13.64 | 0.01 | 0.01 | 28.18 |
| **G** | 17.27 | 0.01 | 0.01 | 0.01 | 0.01 | 0.01 | 0.01 | 0.01 |
| **T** | 9.10 | 0.01 | 0.01 | 0.01 | 46.36 | 99.98 | 64.54 | 35.45 |
