# Supplementary material for: The Drosophila Translational Control Element (TCE) Is Required for High-Level Transcription of Many Genes That Are Specifically Expressed in Testes
Source: PLoS One. 2012 Sep 11;7(9):e45009. doi: 10.1371/journal.pone.0045009 (PMC3439415; doi:10.1371/journal.pone.0045009)
Supplement: Table S3 — qPCR of testis-specific genes relative to actin. (DOC) [file pone.0045009.s003.doc]

**Table S3. qPCR of testis-specific genes relative to *actin***

| **Gene** | **Testes** | **S2 cells** |
| --- | --- | --- |
| *Mst84Dc* | 32.081 ± 1.382 | 0.0000 ± 0.0000 |
| *Mst87F* | 15.677 ± 1.465 | 0.0001 ± 0.0000 |
| *CG7557* | 1.228 ± 0.109 | 0.0001 ± 0.0000 |
| *CG14305* | 0.891 ± 0.143 | 0.0064 ± 0.0020 |
| *CG31245* | 0.572 ± 0.079 | 0.0002 ± 0.0000 |
| *hsp70* | 0.310 ± 0.155 | 0.2152 ± 0.0525 |
