# Supplementary material for: The Drosophila Translational Control Element (TCE) Is Required for High-Level Transcription of Many Genes That Are Specifically Expressed in Testes
Source: PLoS One. 2012 Sep 11;7(9):e45009. doi: 10.1371/journal.pone.0045009 (PMC3439415; doi:10.1371/journal.pone.0045009)
Supplement: Table S4 — Primer sequences (5′-to-3′). (DOC) [file pone.0045009.s004.doc]

**Table S4**. Primer sequences (5’-to-3’)

| **Gene** | **Primer** |
| --- | --- |
| *actin 5C* | CGAAGAAGTTGCTGCTCTGGTTGTCG  GGACGTCCCACAATCGATGGGAAG |
| *β-gal* | CGCCAGTCAGGCTTTCTTTCACAG  GGTCGCTTCACTTACGCCATTGTC |
| *CG7557* | AAAGATACTTTCTGCAGTCCCGAGGC  TTCACACCGCATTCGGAAAGTCCA |
| *CG14305* | CAGTTGGGTACTCGAAGTTCGGATG  GCCGATCTTGTGACCCACATTGTA |
| *Mst84Dc* | TGCTGCGGATATTACTGCTGTGGT  AACTGCCAAATGGTCCGCAACAAG |
| *Mst87F* | GGACCCTGTGGACCCTGCTGCGGA  TGCAACTGTCTAGCAGCAAGGGTA |
| *CG31245* | TCGAGACCCAAGTCATCGATACCCAA  TTGTGGAAGTTGGCCCACTCATTC |
| *hsp70* | TTGGACGAGGCTGACAAGAACTCC  TTGTGGTCGAACTCCTCCTTCTCG |
